# Changing the Focus to the Whole Patient instead of One Oral Disease: The Concept of Individualized Prevention

**DOI:** 10.1155/2020/6752342

**Published:** 2020-05-20

**Authors:** Gerhard Schmalz, Dirk Ziebolz

**Affiliations:** Department of Cariology, Endodontology and Periodontology, University of Leipzig, Leipzig, Germany

## Abstract

Oral diseases are highly prevalent and a global burden. Accordingly, their prevention appears essential. Recently, different strategies have been developed, mainly focusing on the presence of singular oral diseases or conditions. This article aims to construct a contemporary concept of individualized preventive care in dentistry whereby the focus is switched from viewing oral health in isolation to viewing the patient as a whole. The basis for individualized prevention measures is the case-oriented profile, including the synthesis of risk- and need-oriented parameters. The risk profile comprises different risk factors within the fields of systemic diseases, medications, and lifestyle that inherently pose a potential risk of complications (e.g., infectious endocarditis) and/or oral diseases (e.g., periodontitis). The needs profile includes factors originating from the aspects of oral diseases, dental restorations/appliances, and dental results with a potential risk of pathogenesis (e.g., the de novo development of caries) and/or the potential progression of oral diseases (e.g., an existing caries lesion). Based on these parameters, the general framework and content of prevention measures, as well as the maintenance interval, should be adapted to the individual patient. The implications of this concept might increase the safety, effectiveness, and efficiency of prevention in dental care. A further area of focus is primary prevention, that is, a focus on the preservation of oral health instead of a disease-related approach. However, clinical validation is needed to prove the benefits of the model presented. Individualized prevention promotes a shift from a disease-focused model to a whole-patient-focused model and provides a potential approach for establishing a contemporary concept for preventive care in dentistry.

## 1. Introduction

Oral diseases are one of the most prevalent diseases in the world, resulting in an enormous global health problem [1]. The incidence of caries, periodontitis, and associated tooth loss leads to a major health and economic burden and impairs the quality of life of affected individuals [1]. The FDI World Dental Federation recently characterized oral health as a synthesis of the absence of diseases and conditions in addition to sufficient physiological and psychological function [2]. Furthermore, caries and periodontitis are chronic and multifactorial with a dynamic nature, including stages of both progression and stagnation [3, 4]. These diseases require lifelong maintenance for the affected patients [5, 6]. Thus, the recovery and/or preservation of healthy oral conditions according to an appropriate prevention strategy seem essential. However, a paradigm shift from invasive treatment to prevention or from a surgical to a medical model must occur [7]. In contrast, current prevention concepts are focused on the presence of individual diseases or conditions, such as periodontitis (supportive periodontal therapy, SPT), caries, tooth wear, and implant (supportive implant therapy, SIT) as well as prosthodontic therapy [5, 6, 8, 9]. Although these concepts are the basis for established and functional strategies, they are focused on individual diseases and potential disease-related risks and do not consider the patient as a whole. Therefore, a personalized prevention strategy based on individual needs and risks should be developed in the field of dentistry [7].

Another aspect that is often insufficiently addressed within dental prevention is the presence of general diseases and conditions. Various links between oral and systemic health are known; e.g., the bidirectional relationship between periodontitis and diabetes has already been well described [10]. However, systemic diseases and conditions may also be related to a risk of systemic complications for patients during and/or after dental therapy and prevention measures. A risk of systemic infection in immunocompromised individuals or the risk of infective endocarditis can be cited as examples [11, 12].

Accordingly, a contemporary understanding of prevention in dentistry must include and combine the aspects of oral disease-specific, general health-specific, and individual patient-specific aspects. Novel concepts must be assessed in different dimensions, such as “effectiveness,” “efficiency,” and “safety,” as required in the context of personalized medicine [13].

This narrative review aims to construct a contemporary, individualized prevention concept, including different aspects of oral and systemic diseases and conditions. The definitions and classifications of risk and need factors presented here should allow the current approach to be adjusted to focus on prevention from the perspective of the patient as a whole and not from the perspective of singular oral diseases or conditions. This paper only focuses on prevention and is not exhaustive but should illustrate a comprehensive concept of individualized preventive care.

## 2. Main Body

### 2.1. Prerequisites of Individualized Prevention

The basis of patient-centered dental care is the understanding of the complex and individual needs, risks, and perceptions of a patient. Before appropriate risk and need classifications can be applied, a sufficient doctor-patient or dentist-patient relationship remains a mandatory prerequisite; this includes the integration of the patient's values and preferences in the context of shared decision-making [14]. This demand is often not fulfilled in dental settings [15] and must therefore be seen as a basis of patient-oriented prevention. Within individual preventive care, particular importance can be seen in the communication between dental teams and patients. This should include participative communication and motivational interviewing by well-educated dental team members [16]. Accordingly, patients' individually perceived needs and concerns are the primary focus of dental care. The presented risk and need classifications support safe and effective individual preventive care in specific patient cases.

### 2.2. Implication of Systemic Factor: Risk-Oriented Prevention

**Figure 1:**
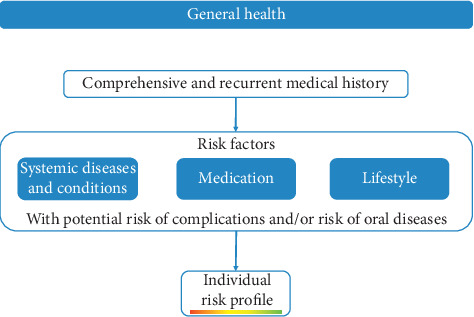
On the basis of a comprehensive and recurrent medical history, risk factors originating from the fields of oral diseases and conditions, medications, and lifestyle lead to the individual risk profile.

The first major aspect of developing individualized preventive measures is the integration of systemic diseases, conditions, medications, and lifestyle into a prevention concept. Within this aspect, the dimensions of “safety” (i.e., the avoidance of complications) and “effectiveness” (i.e., a consideration of the links between oral and systemic health) should be addressed. A *risk factor*, which is defined by the World Health Organization (WHO) as an “attribute, characteristic or exposure of an individual that increases the likelihood of developing a disease or injury” [17], provides the basis for the risk-oriented adjustment of prevention measures. These risk factors can include two different subaspects: a *risk of complications* and a *risk of oral diseases*. The risk of complications includes the increased likelihood of systemic complications during or following prevention measures, e.g., the risk of infectious complications (endocarditis and sepsis) after professional tooth cleaning. The risk of oral diseases is the increased likelihood of the occurrence of an oral disease or condition caused or influenced by a general health condition. The increased risk of periodontitis in patients suffering from different general diseases, such as diabetes or rheumatoid arthritis, can be considered in this context [10, 18–22]. Risk factors must be assessed within a comprehensive and recurrent medical history. In general, risk factors can originate from three different areas, while each risk factor can include a risk of complications and/or oral diseases. The sum of all risk factors constitutes the patient's individual *risk profile* (Figure 1).

#### 2.2.1. Systemic Diseases and Conditions

**Figure 2:**
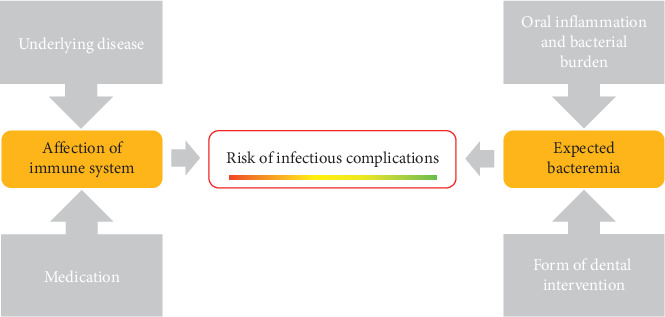
The risk of infectious complications and thus the recommendation of antibiotic prophylaxis can be determined by considering both the effect of the immune system and the level of expected bacteremia.

Different systemic diseases can be seen as risk factors. In the context of the risk of complications, on the one hand, the risk of systemic infections related to bacteremia caused by prevention measures exists [12, 23–25]. This covers, inter alia, an endocarditis risk (e.g., heart valve replacement), insufficiently controlled diabetes, or newly inserted joint prostheses [11, 26, 27]. For these patients, antibiotic prophylaxis, with regard to the extent of immunosuppression (number and effective strength of immunosuppressive drugs and the reason for immunosuppressive medication) and the expected bacteremia, is necessary (Figure 2). On the other hand, the risk of complications directly related to prevention measures is relevant. For example, the avoidance of aerosol formation in the care of patients with chronic obstructive pulmonary diseases is recommended [28].

Moreover, the risk of oral diseases can be influenced by systemic diseases and conditions. Depending on glycemic control (HbA1c: <7% or ≥7%), diabetes mellitus has an influence on periodontitis; accordingly, diabetes mellitus is a parameter for the grading matrix of periodontitis [17, 29, 30]. This well-known example highlights the potential of a patient's systemic health to influence his or her risk of oral diseases, independent of his or her current oral status, and emphasizes the need for increased attention to systemic health to maximize prevention (e.g., maintenance interval 3–4 months).

#### 2.2.2. Medications

Alongside, or combined with, systemic diseases and conditions, the intake of different medications entails potential risk factors. For example, systemic infections within the potential complications can occur in relation to immunosuppressive medications [31]. Another example is the development of jaw osteonecrosis related to bisphosphonate intake [32]. According to several systemic diseases, the intake of these medications can require antibiotic prophylaxis prior to professional preventive measures. Furthermore, there are several medications that influence the risk of oral diseases. Here, the occurrence of gingival overgrowth associated with calcium-channel blockers, phenytoin, or cyclosporine A [33], as well as xerostomia, e.g., related to antihypertensive medication, are of relevance [34]. Independent of oral status, patients who take these medications need increased preventive care.

#### 2.2.3. Lifestyle

Lifestyle choices contribute to the complexity of different risk factors within risk-oriented prevention. However, lifestyle parameters rarely affect the risk of complications. Alcohol and/or drug abuse can influence the immune system and might be related to complications during dental therapy and prevention [35]. However, an increase in the risk of oral diseases is obvious, especially considering that smoking is a key risk factor for periodontitis. This factor is also displayed in the grading matrix for periodontitis, from which, depending on the number of cigarettes, a moderate (<10 cigarettes/day) or high risk of progression (≥10 cigarettes/day) can be derived [29, 30]. Therefore, smoking also represents a risk factor independent of the current oral health situation, resulting in the necessity of increased preventive care. Moreover, nutrition, e.g., veganism, can be seen as a potential risk factor, as it can be related to nutrient deficiency [36]. Additionally, oral piercings are a factor that has the potential to increase the risk for the decay of dental hard and gingival/periodontal soft tissues [37].

### 2.3. Implication of Oral Conditions: Need-Oriented Prevention

**Figure 3:**
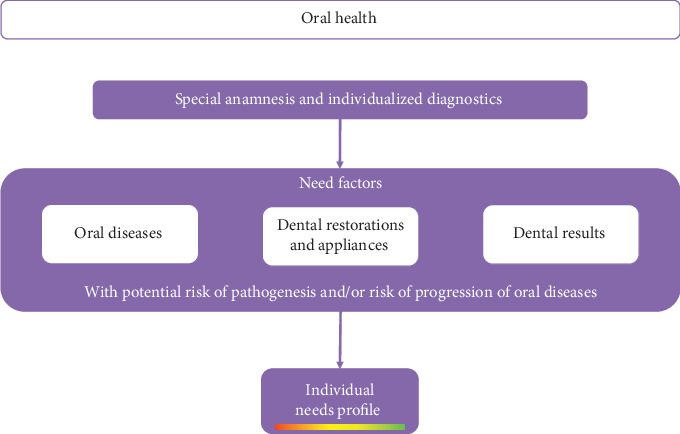
Based on a special anamnesis and individualized diagnostics, need factors originating from existing oral diseases, dental restorations, and appliances as well as dental findings determine patient needs profiles.

Similar to the risk factors within risk-oriented prevention, oral conditions include specific need factors. These need factors describe oral health-related conditions, which can include both a risk of pathogenesis and a risk of oral disease progression. The risk of pathogenesis describes the increased likelihood of the de novo development of an oral disease due to a specific need factor. The origin of root caries on the exposed root surfaces of periodontally diseased teeth is an example of an increased risk of pathogenesis [38]. The risk of progression can be defined as the increased likelihood of the advancement of an oral disease that is already present. This risk of progression is the recent primary target of maintenance, such as the preservation of stable periodontal conditions in patients with a history of periodontitis by supportive periodontal therapy [5]. Within the need aspect, the dimensions of “efficiency” (i.e., avoidance of unnecessary prevention measures) and “effectiveness” (i.e., choice of the most effective prevention measures on an individual basis) should be addressed [13]. Analogous to the risk profile, the sum of all need factors leads to the individual needs profile of the patient (Figure 3). A special anamnesis of oral health behavior and appropriate, comprehensive, and individualized diagnostics form the basis for the detection of need factors. These need factors can originate from three different fields, as described below.

#### 2.3.1. Oral Diseases

This field includes the patient's oral health history. Primarily, the preservation of oral health, as defined by the FDI [2], should be the main goal of dental care. Therefore, even orally healthy individuals require individualized preventive measures because dentistry applications have the additional function of enabling the primary prevention of oral diseases [7]. For an appropriate interpretation of the oral disease burden and the respective risk of pathogenesis and progression, available disease classifications and risk assessments should be utilized. Accordingly, for dental caries, the International Caries Detection and Assessment System (ICDAS-II) [39, 40] in combination with an adequate caries risk assessment [41] should be used. The ICDAS-based caries activity combined with the caries risk provides information about the risk of the progression of existing carious lesions. Moreover, the caries risk assessment allows conclusions to be drawn regarding the risk of pathogenesis for new lesions.

Similarly, the new classification of periodontal diseases and conditions with a staging and grading matrix describing the stage of the periodontal burden as well as the risk of progression [29, 30], complemented by a periodontitis risk assessment [42], allows for needs assessments of individuals with periodontal diseases. Periodontal health and gingival diseases on intact or reduced periodontium should be considered accordingly [43]. While periodontitis grading and risk assessments provide information on the risk of progression, the risk assessment and presence of gingivitis are informative regarding the risk of periodontitis pathogenesis [44].

The complexity becomes clear when caries and periodontitis are linked within the needs profile: a higher stage of periodontitis can be related to an increased risk of pathogenesis for root caries on exposed dentin areas [38]. In addition to these two common oral diseases, other oral health parameters, such as tooth wear (erosion) or oral mucosal diseases, including potentially premalignant lesions, must be recognized [9, 45].

#### 2.3.2. Restorations and Appliances

Different aspects of need-oriented prevention are related to the presence of dental restorations, prosthodontic treatment, or appliances in the oral cavity. Tooth-borne restorations (fixed and/or removable) require a maintenance regime [8], as there is a risk of pathogenesis, especially for caries at the restorative margin. The preventive care of implants seems even more complex, even though supportive implant therapy as a special care concept for dental implants exists [4, 46, 47]. Within this maintenance therapy approach, different patient-specific (e.g., oral hygiene behavior and smoking) and implant-related parameters (e.g., the presence of keratinized mucosa and design of suprastructure) as well as specific diagnostic findings (e.g., bleeding/suppuration on probing, radiographic bone loss) are of relevance [48]. The main issue in the supportive care of implants is to preserve peri-implant health, which recently received a consistent definition for the first time [49]. The risk of pathogenesis for peri-implantitis can be related to the number of implants, the complexity of the restoration, and the presence of risk factors such as smoking or history of periodontitis [50–53]. The risk of progression of peri-implantitis is additionally determined by the presence of mucositis, especially in the case of suppuration and an increase in probing depth [46]. Furthermore, dental implants require specific prevention measures, such as the use of suitable instruments for professional cleaning to avoid damage to implant surfaces [54]. Fixed orthodontic appliances are another example associated with an increased risk of pathogenesis for caries and periodontal diseases [55, 56].

#### 2.3.3. Dental Results

This field has the largest dynamic within the needs profile and represents the diagnosis following a clinical assessment of oral tissues. However, the targeted assessment of dental and periodontal findings as well as oral hygiene indices are the most important measures for adequately estimating the risk of pathogenesis and the progression of oral diseases. In particular, the accumulation of dental biofilm (plaque) on tooth surfaces and gingival inflammation must be seen as important parameters in the etiopathogenesis of caries and periodontitis [57, 58]. Moreover, these parameters allow the compliance and oral health behavior of patients to be assessed and are therefore mandatory for preventive care [59]. The form and extent of necessary diagnostic examinations are determined by the oral disease history (see Section2.3.1) and the presence of tooth- or implant-borne restorations or appliances (see Section 2.3.2) and should be adapted to the individual patient at each appointment.

### 2.4. The Synthesis of Risk and Need Profile: the Case-Specific Patient Profile

**Figure 4:**
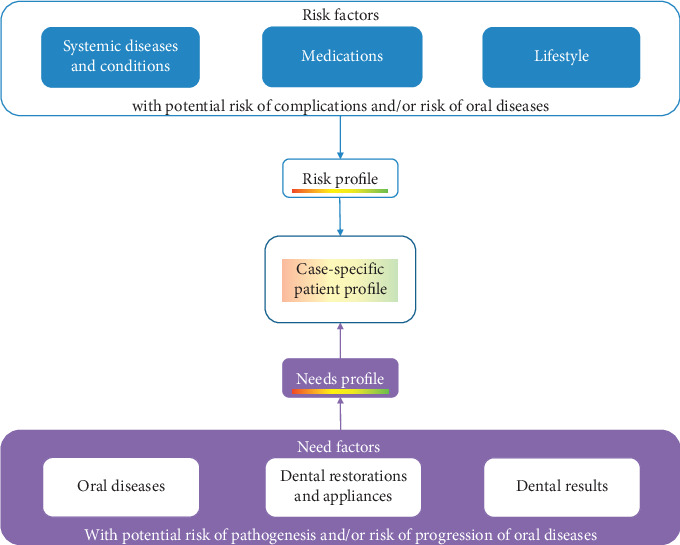
The synthesis of a risk and needs profile leads to the case-oriented patient profile, which determines the general framework, content, and frequency of the prevention cycle for the individual patient.

**Figure 5:**
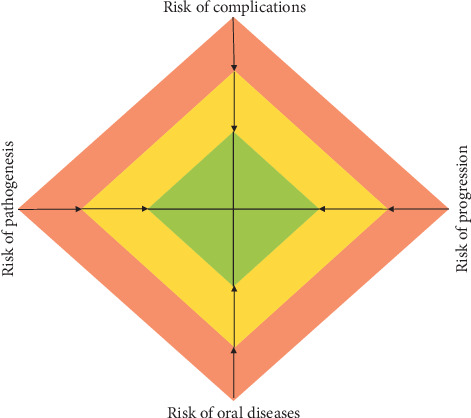
For each patient, an individual risk in the four domains can be evaluated. The vertical dimension can be seen as the “risk axis,” and the horizontal dimension describes the “need axis.” Based on these axes, the individual prevention regimen for each patient can be evaluated.

The synthesis of risk-oriented and need-oriented prevention into one model is necessary to fulfill the demand for a contemporary, individualized prevention concept. Consequently, the four risks defined above, including the risk of complications and risk of oral disease within specific risk factors as well as the risk of pathogenesis and risk of progression within corresponding need factors, lead to a case-specific patient profile (Figure 4). To allow practical implementation, an appropriate risk classification should be added as a transferable basis for the risk and needs profile. In this way, each risk can be classified into one of three classes: low, moderate, or high risk (Table 1). The applied risk-/need-classes are not always a clear binary choice because several risks show a dynamic transition between moderate and high risks. Moreover, the moderate class can be quite heterogeneous. In each case, the highest class is decisive for classifying the patient as a low-, moderate-, or high-risk individual. This categorization might allow for the rapid and efficient allocation of the patient. Moreover, the classes can be translated into a clinical consequence. Low risk indicates no consequences for the general framework and main content of the prevention cycle. These patients only require “basic prevention” to preserve their stable/healthy conditions, e.g., once a year. For individuals in the moderate-risk class, the general framework and content of the prevention measures should be adapted according to their risk and need. Thus, general health risks should be considered, and oral health-specific conditions should be recognized. The maintenance interval has to be adapted to the individual patient and often levels out at 6 months. For patients in the high-risk class, risk management, that is, the adaptation of the general framework (e.g., antibiotic prophylaxis) and content of the prevention cycle is necessary for patient safety and/or therapy success. These patients regularly need highly frequent prevention at an individual interval between 3 and 4 months. Within this construct, the case-specific patient profile allows the classification of low-, moderate-, or high-risk patients with the related consequences. The focus is on the whole, individual patient and not on the presence of singular oral diseases or conditions (Figure 5). Although a clear clinical consequence cannot be derived from the respective risk class in every case, two factors offer potential clinical benefits. First, placing a patient in the moderate category could be seen as a preparatory step for the special conditions of the case. The second important issue is the support for communication about oral health-related conditions and preventive measures between dental teams and patients.

## 3. Discussion

The concept of individualized prevention as presented in this manuscript combines patient-specific risk- and oral-health-specific needs profiles and shifts the focus from oral diseases/tooth-related parameters to the whole patient. The joint prevention of different oral diseases, including caries and periodontitis, considered together is not, however, a new approach [6, 60]. Moreover, risk assessment, as seen regarding the risk of periodontitis, is already based on patient-related factors such as diabetes mellitus or smoking habits [20, 42]. Accordingly, the available prevention strategies include parts of the described concept of individualized prevention, but until now, they have failed to combine the different aspects from the perspective of the patient as a whole. The number of general health problems is growing worldwide, whereby noncommunicable diseases are an emerging global burden [61]. Especially in the elderly population, general health conditions with potential interrelationships with oral diseases are an issue of increasing practical relevance [62]. Therefore, the aspect of a related general risk of complications during and/or following preventive measures is not explicitly included in previous prevention concepts. However, there are many patient-specific factors that potentially influence the general framework of prevention (e.g., antibiotic prophylaxis prior to prevention measures). These include an infectious risk for endocarditis (e.g., heart valve replacement and history of endocarditis) or other systemic infectious complications in immunocompromised patients after organ transplantation, patients experiencing immunosuppression due to autoimmune disease (e.g., rheumatoid arthritis), patients undergoing dialysis, and patients with insufficiently controlled diabetes mellitus or infectious diseases, , e.g., HIV/AIDS [11, 26, 31, 63–65]. Furthermore, the content of prevention must be adapted based on the patient's risk of complications. In this context, the avoidance of aerosol formation in patients with respiratory diseases [28], avoidance of several ultrasonic devices in patients with pacemakers [66], or avoidance of adrenaline-containing devices in patients with glaucoma [67] can serve as examples. Considering these factors with respect to the risk of complications seems essential to ensure treatment safety. Accordingly, this consideration addresses the dimension of “safety” as previously defined [13] and may therefore provide an improvement over available concepts. However, the practical benefit remains hypothetical, and validation in a clinical setting is needed.

Furthermore, some of the diseases and conditions listed above also include a risk of oral diseases. Oral diseases, especially caries and periodontitis, are multifactorial [3, 4]. Available risk assessments do consider this factor but are primarily focused on sugar intake and salivary flow for caries or smoking and diabetes mellitus for periodontitis [20, 41, 42]. However, the risk factors for oral diseases are more multifarious and complex. Rheumatic diseases [21, 22], osteoporosis [68], renal insufficiency [69], radiation therapy [70], infectious diseases [63], and many medications causing oral side effects [33, 34] can affect the occurrence of caries and/or periodontitis. A problem of growing importance is the worldwide increase in obesity, which has a clinically relevant relation to periodontal inflammation; obese patients have special needs regarding dental care within a multidisciplinary concept [71]. Consideration of these oral disease risk factors and the related individual adaptation of prevention measures seem essential to ensure therapeutic success. Accordingly, this aspect should address the dimension of “effectiveness” [13] and might confer a benefit compared to existing concepts. Implementation of the individual risk profile for prevention can fulfill the demand for a necessary shift from a solely surgical model to a contemporary medical model of dental preventive care [7]. However, a comprehensive and recurrent medical history, as well as an interdisciplinary collaboration between dentists and general physicians, appears necessary to accomplish this part of individualized prevention in practice. This is of particular importance in patients with complex general conditions, e.g., in elderly patients. These patients show different particularities, including xerostomia, physical and/or cognitive limitations, and, thus, particularities regarding their ability and willingness to implement preventive recommendations [72, 73]. Moreover, differences in the views of dentists and general physicians could be a limitation in terms of the appropriate practical implementation of risk-oriented prevention [74]. Nonetheless, the consideration of lifestyle choices, including smoking (duration and quantity), nutrition (sugar consumption, intake of vitamins, and nutrients) and oral hygiene behavior (form and frequency of tooth-brushing, interdental cleaning, and fluoride usage), still constitutes a keystone of prevention and must be recognized and adapted in a patient-specific manner [7]. Accordingly, these lifestyle parameters complete the patient's risk profile.

The second main aspect of the individualized prevention concept is that of the patient's needs profile. Recent strategies have primarily focused on arresting/stabilizing disease processes such as caries and/or periodontitis and halting the progression of the existing oral disease [5, 6]. Although this approach is both effective and efficient, it displays three weak points. First, it mainly addresses the risk of progression and thus represents only a secondary prevention approach to the diseases, as explained by Birch et al. [7]. Second, individuals with good oral health are not considered. While the preservation of the entire complex nature of oral health, including the absence of diseases and conditions, and sufficient physiological and psychological function [2] should be emphasized, at the same time, individualized (depending on risk of pathogenesis) prevention for patients without any history of oral disease might be necessary. Therefore, case-specific, probably individualized “basic prevention” once a year or once every two years should also be applied to these individuals as a primary prevention measure. Third, the complexity of patients' need factors, originating from the characteristics of oral diseases, restorations/appliances, and/or recent dental findings and their potential mutual influence, must be addressed. This seems hardly possible when only singular diseases are the focus of preventive measures. These points are addressed by the construct of need-oriented prevention. Clinical assessments of the individual's needs, including the risk of pathogenesis and/or progression of oral diseases, do not have to be reinvented. In fact, available classifications and risk assessments should be applied in combination, depending on each patient. For example, concerning oral diseases, the recent classification for periodontal diseases and conditions in combination with the established periodontal risk assessment [29, 30, 42], and for caries, the ICDAS-II complemented by caries risk-assessment [39–41] should be combined in respective clinical situations. The risk of pathogenesis caused by a mutual influence, for instance, the risk of root caries on exposed dentin caused by gingival recession due to periodontal history [38], should be recognized. This field is complemented by the consideration of dental restorations or appliances, including tooth- and implant-borne restorations, as well as orthodontic treatment with the related risks of pathogenesis and/or oral disease progression [8, 55, 75].

The dynamic aspect within the needs profile is the assessment of current dental findings. Basic diagnostics (anamnesis and dental and periodontal findings), especially including oral hygiene indices, should be applied [57, 58]. Depending on the oral disease history and restorations/appliances, specific examinations should be supplemented, e.g., a comprehensive periodontal diagnosis (probing depth, bleeding on probing, and clinical attachment loss) in patients with periodontal history or upon probing near dental implants combined with indication-based radiographs [5, 76]. Within this field, additional diagnostic procedures might become relevant in the future. In the context of personalized medicine in dentistry, the consideration of individual genetic risk indicators could be applied [77]. Additionally, epigenetic markers, such as noncoding RNAs, would be a potential future perspective [78]. Furthermore, although their recent diagnostic benefit for prevention has not been completely clarified, matrix metalloproteinase in gingival crevicular fluid or in saliva could be promising [79, 80]. The consideration of an individual needs profile, with a differentiation between the risk of pathogenesis and the risk of oral disease progression, primarily addresses the dimension of the “effectiveness” of the concept. The most effective preventive measure might be related to the respective need and should lead to the avoidance of disease development and/or pathogenesis.

**Figure 6:**
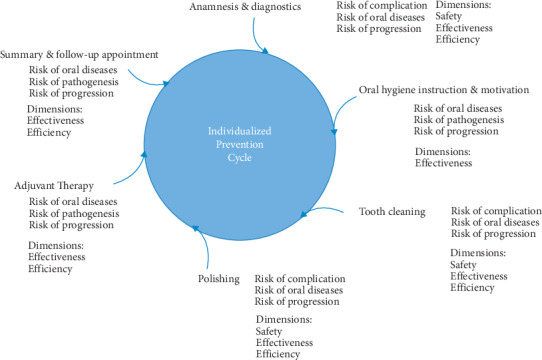
The practical implication of the concept of individualized prevention. Each basic element of the prevention session is influenced in its content and general framework by the four different risks within the case-oriented profile. The main risks within the six basic elements are presented alongside the primary related dimensions.

Furthermore, the dimension of “efficiency” can also be recognized because unnecessary diagnostic and prevention measures might be avoided with a case-oriented adaptation of the prevention cycle. Prevention in dental practice should follow a structured setup [57]. Each session should include anamnesis/diagnostics, oral hygiene instruction and motivation, tooth cleaning, polishing, adjuvant therapy, and follow-up appointment planning. These basic elements are determined by the different risks within the case-oriented profile and need to be adapted to the individual patient (Figure 6). The resulting individual prevention cycle for each patient and session is the practical consequence of the described construct. This individualized cycle is a foundation for the content and conditions of the preventive measures; however, the individual concerns of the patient must be recognized, and a respective adaption of parts of the prevention cycle according to patient's preferences should be made. Therefore, the basic principles of shared decision making must be considered [14].

This manuscript primarily focused on the singular consideration of different risk factors; some of these factors might be additive, but they may also be synergistic, influencing health or diseases more than the added weight. Accordingly, available risk models combining different indicators should be considered for future risk assessment and classification approaches. These factors could include, e.g., the interrelation between cardiovascular and periodontal health with a modified risk ratio affected by smoking, diabetes, C-reactive proteins, interleukins level, and other factors [81], as well as patient-centered risk assessments in implant therapy [82].

However, several limitations can be listed. On one hand, cost-effectiveness is an important topic related to maintenance therapy [83, 84]. The cost-effectiveness, especially of the primary prevention of orally and systemically healthy individuals, can be discussed but might be seen in the context of ethical issues as medical responsibility. On the other hand, validation of the potential benefits of the model presented here is still absent. Between different countries, insurance schemes might still be limiting factors for care that is primarily focused on prevention; the provision of preventive advice is often either dismissed or poorly remunerated in comparison to the provision of invasive restorative treatment. This can be an important barrier to change from a treatment-oriented oral care system to one in which the emphasis is on individualized prevention. Based on this article, the benefits are discussed hypothetically and require verification in a clinical setting. Moreover, consideration of all the aspects from the risk and need profile within the case-oriented profile might be challenging for dentists and dental assistants, e.g., dental hygienists. Therefore, training courses and support for the practice would be necessary. This concept only addresses the context of dental prevention or preventive measures. Further issues might be of relevance for invasive dental treatment procedures, which are not mentioned within this review article. Additionally, different major aspects of prevention as a contemporary concept, including primordial prevention, collective prevention, and biopsychosocial considerations of patient-as-a-person situations, are not exclusively addressed within this manuscript. These issues must be recognized and addressed appropriately. Moreover, the three applied risk classes (low, moderate, and high) are limited; in several cases, a clear binary choice is possible (e.g., the presence or absence of endocarditis prophylaxis indicating a high- or low-risk class, respectively). In contrast, several cases show a dynamic transition between moderate and high risks (e.g., immunosuppressive medication can vary between moderate- or high-risk classes depending on the number and effective strength of the immunosuppressive drugs). Accordingly, the classification must be seen as a guiding support, always making an individual clinical consequence necessary. The classification might increase the sensitization of patients and dental teams to different issues (systemic and oral health concerns) and could support the instruction, awareness, and motivation of the patient. However, this remains speculative and needs further evaluation.

## 4. Conclusions

A contemporary, individualized prevention concept for dental maintenance should recognize the whole patient within a case-oriented profile. At the same time, the risk of complications and/or oral diseases originating from systemic diseases, medications, and lifestyle choices must be included. Furthermore, the risk of pathogenesis and/or progression of oral diseases based on the assessment of existing oral diseases, dental restorations/appliances, and recent dental results must be determined. This might allow safe, effective and efficient prevention based on the patient's individual risks and needs within an individual prevention cycle. Clinical evaluation of the described construct is necessary to prove its practical benefits.

## Figures and Tables

**Table 1:** Definitions and examples for the different risk classes within the risk and needs profiles.

Class		Risk profile		Needs profile	
		Risk of complications	Risk of oral diseases	Risk of pathogenesis	Risk of progression
Low	Definition	No increased risk of complications	No increased risk of oral disease	No increased risk of de novo oral disease development	No increased risk of the progression of existing oral diseases
	Example	Overall healthy patient	Overall healthy patient	Caries/periodontitis risk: low	Orally healthy patient

Moderate	Definition	Moderately increased risk of complications	Moderately increased risk of oral diseases	Moderately increased risk of de novo oral disease development	Moderately increased risk of the progression of existing oral diseases
	Example	Well controlled COPD, bronchial asthma	Well controlled diabetes mellitus (HbA1c < 7%)	Caries/periodontitis risk: moderate	Active ICDAS 1-2 combined with a low/moderate caries risk, periodontal health at reduced periodontium

High	Definition	High risk of complications, potentially life-threatening risk	High risk of oral diseases	High risk of de novo oral disease development	High risk of the progression of existing oral disease
	Example	Heart valve replacement	Insufficiently controlled diabetes mellitus (HbA1c > 7%)	Caries/periodontitis risk: high	Active ICDAS combined with high caries risk, periodontitis grade C

ICDAS: International Caries Detection and Assessment System; COPD: chronic obstructive pulmonary disease.
